# Biochemical Characterization of Putative Adenylate Dimethylallyltransferase and Cytokinin Dehydrogenase from *Nostoc* sp. PCC 7120

**DOI:** 10.1371/journal.pone.0138468

**Published:** 2015-09-16

**Authors:** Jitka Frébortová, Marta Greplová, Michael F. Seidl, Alexander Heyl, Ivo Frébort

**Affiliations:** 1 Department of Chemical Biology and Genetics, Centre of the Region Haná for Biotechnological and Agricultural Research, Faculty of Science, Palacký University, Olomouc, Czech Republic; 2 Department of Molecular Biology, Centre of the Region Haná for Biotechnological and Agricultural Research, Faculty of Science, Palacký University, Olomouc, Czech Republic; 3 Laboratory of Phytopathology, Wageningen University, Wageningen, The Netherlands; 4 Institute of Biology/Applied Genetics, *Dahlem Centre of Plant Sciences*, Freie Universität Berlin, Berlin, Germany; Universidade Federal de Vicosa, BRAZIL

## Abstract

Cytokinins, a class of phytohormones, are adenine derivatives common to many different organisms. In plants, these play a crucial role as regulators of plant development and the reaction to abiotic and biotic stress. Key enzymes in the cytokinin synthesis and degradation in modern land plants are the isopentyl transferases and the cytokinin dehydrogenases, respectively. Their encoding genes have been probably introduced into the plant lineage during the primary endosymbiosis. To shed light on the evolution of these proteins, the genes homologous to plant adenylate isopentenyl transferase and cytokinin dehydrogenase were amplified from the genomic DNA of cyanobacterium *Nostoc* sp. PCC 7120 and expressed in *Escherichia coli*. The putative isopentenyl transferase was shown to be functional in a biochemical assay. In contrast, no enzymatic activity was detected for the putative cytokinin dehydrogenase, even though the principal domains necessary for its function are present. Several mutant variants, in which conserved amino acids in land plant cytokinin dehydrogenases had been restored, were inactive. A combination of experimental data with phylogenetic analysis indicates that adenylate-type isopentenyl transferases might have evolved several times independently. While the *Nostoc* genome contains a gene coding for protein with characteristics of cytokinin dehydrogenase, the organism is not able to break down cytokinins in the way shown for land plants.

## Introduction

Cytokinins are a group of plant hormones affecting various aspects of plant development and the response to abiotic and biotic changes in the environment [1,2]. The biosynthesis of cytokinins starts with a transfer of dimethylallyl chain from dimethylallyl pyrophosphate (DMAPP) or *(E)*-4-hydroxy-3-methyl-but-2-enyl diphosphate (HMBPP) to *N* ^6^-amino group of free or tRNA-bound adenosine phosphates [3–5]. The reactions are catalyzed by related but distinct enzymes, adenylate dimethylallyltransferase (EC 2.5.1.27) and tRNA dimethylallyltransferase (EC 2.5.1.75), respectively, more frequently termed as isopentenyl transferases (IPT). In modern land plants, the major share of cytokinins is synthesized by adenylate IPTs, present as multiple isoenzymes, while the release of tRNA-bound cytokinins due to tRNA degradation is considered as minor biosynthetic pathway [6]. The tRNA pathway however appears to be exclusively responsible for biosynthesis of *cis*-zeatin type cytokinins modern plants [7]. Early diverging land plants were thought to use only the tRNA pathway for cytokinin production [8], but a recent mutant analysis in the moss *Physcomitrella patens* hints to the existence of an alternative tRNA-independent pathways for cytokinin production in those plants [9]. Many bacteria are also able to produce cytokinins [10], but only few of them were shown to contain adenylate IPT [11–19]. In other bacteria, the turnover of modified tRNA is thought to be the sole cytokinin source. This has for example been shown for *Methylobacterium* spp., in which tRNA is source of *trans*-isomer of zeatin [20]. Despite the fact that many bacteria were shown to produce cytokinins, there is only little known about their function in these organisms. Some exceptions are plant pathogens, such as *Rhodococcus fascians*, in which cytokinins function in development of leafy galls [15], or human pathogen *Mycobacterium tuberculosis*, which secretes cytokinins that are involved in sensitization towards nitric oxide [21].

Cytokinins can be inactivated or degraded by a number of different pathways [2]. A key protein regulating the concentration of active cytokinins in plants is cytokinin dehydrogenase (CKX; EC 1.5.99.12) catalyzing an irreversible, oxidative cleavage of cytokinin side-chain [22,23]. Land plants generally contain multiple CKX proteins, with distinct tissue and subcellular localization and substrate specificity [24–28]. Genes homologous to plant *CKX* genes were identified in several bacteria [2,29], but to date only CKX from *R*. *fascians* has been characterized in more detail and shown to possess CKX activity [30].

While it has been proposed that the cytokinin regulatory system was introduced into the plant lineage via horizontal gene transfer during endosymbiosis [31], evidence for this hypothesis is not clear. Although most of the protein domains crucial for cytokinin signaling in plants are present in cyanobacteria, they are not assembled in such a fashion as to serve in the signaling of the phytohormone [32]. The picture for the cytokinin metabolism is even less clear. Key enzymes for cytokinin synthesis and degradation (adenylate IPT and CKX) are found only in few species of cyanobacteria [2, 9]. Therefore, we aimed in this study to test *in vitro* if representatives of these two key genes of cytokinin metabolism, an *IPT* and a *CKX* from *Nostoc* sp. PCC 7120, encode proteins that can function biochemically like their homologues in land plants. This should provide some clues towards the question if cyanobacteria use the same mechanism to control cytokinin homeostasis as land plants and therefore further extend our understanding of the evolution of this plant hormone. We selected the cyanobacterium *Nostoc* sp. PCC 7120 as a model species as it is not involved in plant pathogenesis and thus its cytokinin metabolizing capacities might represent an original feature of cyanobacteria rather than an adaptation to the lifestyle of plant pathogens such as *R*. *fascians*. The respective genes, *NoIPT1* and *NoCKX1*, were cloned, expressed in *E*. *coli* and the resulting proteins functionally characterized. More studies including other species are needed to elucidate the function of cytokinins in cyanobacteria in general.

## Materials and Methods

### Cytokinin analysis

*Nostoc* sp. PCC 7120 was cultivated for 28 days in nitrogen-free medium BG-11_0_ [33], in a chamber (Sanyo MLR 350H, Osaka, Japan) with 16 h light/8 h dark cycles, at 24°C and a photon flux density of 35 μmol/m^2^s. Humidity was kept at 60%. The cells were harvested by centrifugation at 20,000 *g* for 15 min at 4°C, washed by deionized water and stored at -20°C. Cytokinins were purified from *Nostoc* cells (0.5 g fresh weight) according to previously described method [34] and subsequently analyzed by UPLC/MS [35].

### Cloning of the *NoIPT1* gene

Genomic DNA was isolated from *Nostoc* sp. PCC 7120 cultivated for 21 days by a method based on a published protocol [36]. The sequence of *NoIPT*1 (locus tag: PCC7120DELTA_RS03075) was amplified from the genomic DNA of *Nostoc* sp. PCC 7120 with the use of Phusion DNA Polymerase (Finnzymes, Espoo, Finland) using primers NoIPT1_NdeI_fw and NoIPT1_SalI_rev (see S1 Table for primer sequences). A TGradient Thermocycler (Biometra, Goettingen, Germany) was programmed as follows: 30 s at 98°C, followed by 35 cycles of 10 s at 98°C, 15 s at 61°C, 30 s at 72°C; and terminated by 10 min at 72°C. *NoIPT*1 gene was further cloned into pET-28b(+) (Merck Millipore, Darmstadt, Germany) vector and the plasmid construct was transformed into *E*. *coli* TOP10 (Invitrogen) by electroporation. Selection of transformants was based on kanamycin resistance and the proper plasmid (confirmed by sequencing) was isolated and transformed into expression cells *E*. *coli* BL21 (DE3) STAR (Invitrogen).

### Production of recombinant *NoIPT1* enzyme in a bacterial expression system

Bacteria were cultivated overnight at 37°C in 20 ml of liquid LB media (kanamycin 50 μg/ml) in a 100 ml Erlenmeyer flask, with orbital shaking at 150 rpm. The bacterial culture was then used to inoculate fresh 50 ml aliquots of the same medium in 250 ml Erlenmeyer flasks (1 ml of inoculum per 50 ml of media). These aliquots were cultivated at 37°C on an orbital shaker (150 rpm) until their OD_600_ value rose to 0.5. Expression of NoIPT1 protein was induced by adding isopropyl-β-D-thiogalactopyranoside to a final concentration of 0.5 mM. Cultures were incubated at 25°C on an orbital shaker (150 rpm) for 5 h, centrifuged at 4,500 *g* to harvest bacterial cells, which were then frozen at -80°C. The pellet from 500 ml of expression culture was suspended in 15 ml of lysis buffer (50 mM Tris/HCl, pH 7.5, 10 mM MgCl_2_, 10 mM β-mercaptoethanol, 10% glycerol) and then disrupted by sonication (3 x 1 min, pulses 5 s). The cell lysates were clarified by centrifugation at 20,000 *g* (+4°C). Subsequent purification consisted of two steps. First, the protein was loaded on High Q column (BioRad, Hercules, CA, USA; 20 cm x 1.5 cm) equilibrated with the lysis buffer. The column was washed by 85 ml of the same buffer and retained proteins were then eluted by a linear gradient of NaCl (0–1 M) in lysis buffer (total volume 175 ml), followed by isocratic elution with 1 M NaCl in the lysis buffer (70 ml). Fractions containing NoIPT1 (as judged from SDS-PAGE and chromatographic data records) were pooled and imidazole was added to reach the concentration of 20 mM. After adjusting pH to 7.5 by HCl, the sample was slowly loaded onto a column filled with 1 ml of HIS-Select Nickel Affinity Gel (Sigma-Aldrich, St. Louis, MO, USA) prewashed with washing buffer A (50 mM Tris/HCl, pH 7.5, 500 mM NaCl, 500 mM KCl, 10 mM MgCl_2_, 10 mM β-mercaptoethanol, 20 mM imidazole). The column was washed by 2 x 1 ml of the washing buffer A, followed by 4 x 2.5 ml of washing buffer B (50 mM Tris/HCl, pH 7.5, 300 mM NaCl, 10 mM MgCl_2_, 10 mM β-mercaptoethanol, 10 mM imidazole) and then eluted by 4 x 1 ml of elution buffer (50 mM Tris/HCl, pH 7.5, 300 mM NaCl, 10 mM MgCl_2_, 10% glycerol, 250 mM imidazole). Eluted NoIPT1 was immediately divided into aliquots, frozen in liquid nitrogen and stored at -80°C.

### Cloning of the *NoCKX1* gene

The sequence of *NoCKX1* (locus tag: PCC7120DELTA_RS03560) was amplified from genomic DNA of *Nostoc* sp. PCC 7120 with the use of Phusion DNA Polymerase. A TGradient Thermocycler was programmed as follows: 30 s at 98°C, followed by 35 cycles of 10 s at 98°C, 15 s at 61°C, 45 s at 72°C; and terminated by 10 min at 72°C. All primer sequences are shown in S1 Table. *NoCKX1* gene was then cloned into various vectors (Table 1), and plasmid constructs were transformed into *E*. *coli* TOP10 by electroporation. Selection of transformants was based on either ampicillin or kanamycin resistance. As expression strains *E*. *coli* BL21(DE3) STAR, BL21(DE3) pLysS (Invitrogen), Origami B (DE3) (Novagen, Darmstadt, Germany) and finally Arctic Express (DE3) (Stratagene, La Jolla, CA, USA) were used. All clones were verified by DNA sequencing.

**Table 1:** List of vectors and *E*. *coli* expression strains used in *NoCKX1* study.

Vector name	Fusion tag	*E*. *coli* expression strain
pQE40 (Qiagen)	6xHis	BL21 (DE3) pLysS
pMAL-c4X (NEB)	MBP	BL21 (DE3) STAR, BL21 (DE3) pLysS, Arctic Express (DE3), Origami B (DE3)
pTYB12 (NEB)	Intein + CBD	BL21 (DE3) STAR, Arctic Express (DE3), Origami B (DE3)
pCIOX^a^	SUMO + 8xHis	BL21(DE3)STAR

^a^pCIOX vector is a modified version of the commercial pET SUMO vector of Invitrogen. It was a kind gift from prof. Andrea Mattevi.

### Production of recombinant *NoCKX1* enzyme in bacterial expression systems

Every transformant culture was diluted to OD_600_ = 0.1, then grown at 37°C in LB medium containing 1% glucose and an appropriate antibiotic until it reached OD_600_ = 0.5 and subsequently induced for 16 h at 18°C with 0.4 mM isopropyl-β-D-thiogalactopyranoside. Arctic Express (DE3) cells were grown at 30°C until OD_600_ = 0.5, then induced for 16 h at 12°C with 1 mM isopropyl-β-D-thiogalactopyranoside. The cells were collected and resuspended in a lysis buffer according to the manufacturer’s protocol and disrupted by a French press (20000 psi) (Thermo, Waltham, MA, USA). The lysate was centrifuged and the supernatant purified by affinity chromatography. His-tagged proteins were purified on Ni Sepharose HP (GE Healthcare, Little Chalfont, UK; 9.5 x 1 cm) or NTA Agarose (Qiagen, Hilden, Germany; 9.5 x 1 cm) and chitin binding domain-tagged proteins on a chitin resin (NEB, Ipswich, MA, USA; 9.5 x 1.6 cm) as published before [37]. To purify proteins fused with maltose binding protein (MBP) an amylose resin (NEB; 9.5 x 1.6 cm) was used. The column was equilibrated in 20 mM Tris/HCl (pH 7.4), 200 mM NaCl and 1 mM EDTA. The protein sample was loaded onto the column and washed with 180 ml of equilibration buffer. NoCKX1 was subsequently eluted with 60 ml of equilibration buffer supplemented with 10 mM maltose. The elution fraction was concentrated on Amicon centrifugal cellulose filter with cut off 10 kDa (Millipore) and stored at -20°C. Maltose binding domain was removed from the purified tagged proteins by the cleavage with Factor Xa (NEB) at 15°C overnight followed by purification on amylose resin. SUMO-fused proteins were treated with SUMO Protease 1 (LifeSensors, Malvern, PA, USA) according to the manufacturer’s manual. Protein content in enzyme samples was determined by the Bradford method [38] with bovine serum albumin as a standard.

### Chaperonin removal

To separate the co-purifying chaperonin, three different procedures were tried. First, the lysate was incubated with 5 mM ATP, 10 mM MgCl_2_ and 150 mM KCl for 1 h at room temperature followed by loading and purification on the amylose column [39]. Alternatively, after the previous incubation step 2.3 M urea was added and the lysate was left for another hour with gentle shaking then dialyzed and purified by column chromatography with chaperonin elution by 2.3 M urea [40]. The last method was applied after protein purification when the eluate was dialyzed overnight at 4°C against 8 M urea, 6 M guanidine-HCl or 1–4% Chaps, respectively. Then the solution was loaded on Superdex 200 10/300 GL (GE Healthcare, Uppsala, Sweden) and gel filtration was performed [41,42].

### Production of recombinant *NoCKX1* enzyme in *Pichia pastoris*

pGAPZαA(His)_10_ and pPICZA(His)_10_ shuttle vectors [37] with zeocin resistance were used for the expression of recombinant NoCKX1 in *Pichia pastoris*. The plasmid construct pGAPZαA(His)_10_::NoCKX1 was linearized with *BlnI* (Takara, Kyoto, Japan) and pPICZA(His)_10_::NoCKX1 with *SacI* (Takara) and used for integration into the *P*. *pastoris* X-33 (Invitrogen) genome. Electroporation at 1.5 kV, transformant selection and protein expression was performed as previously described [37]. The CKX activity was assayed in the cell-free medium or cell lysate, respectively, as described later.

### Overexpression of *NoCKX1* in tobacco

The *NoCKX1* gene from previously prepared plasmid constructs was inserted into the pBin-HYG-TX vector downstream of the 35S promoter. In addition, *NoCKX1* gene was fused with an oligonucleotide coding for apoplastic N-terminal signal sequence from AtCKX2 protein [43] or a vacuolar signal sequence from AtCKX1 [37]. The final vectors were electro-transformed into *Agrobacterium tumefaciens* GUS3101 and all three constructs were used for further transformation of tobacco plants.

*Nicotiana tabacum* leaves were aseptically cut into 1 cm discs and submerged for 20 minutes in a culture of *A*. *tumefaciens* bearing the respective vector, grown overnight in Luria broth at 28°C and re-suspended in Murashige-Skoog (Sigma-Aldrich) medium to OD_600_ = 0.6. Subsequently, the discs were blotted dry and incubated upside-down on MS plates containing 0.7 mg/l benzyladenine and 0.1 mg/l 1-naphthaleneacetic acid for induction of calli regeneration. The plates were placed in an environmental chamber with the photoperiod of 24°C/16 h/light and 22°C/8 h/dark, at a photon flux density of 270 μmol/m^2^s. After 2 days the discs were transferred on a fresh MS medium that in addition contained antibiotics to inhibit bacterial growth, 15 mg/l hygromycin B and 1g/l timentin. Well-developed shoots were rooted on the hormone-free MS medium containing hygromycin B and timentin. Plantlets with a developed root system were placed in soaked jiffy pellets (A/S Jiffy Products, Norway) and grown in a greenhouse with 15 h light/9 h dark cycles, at 25°C. After an acclimatization phase the plants were transferred to soil. Leaf material was harvested before flower induction, frozen immediately in liquid nitrogen and stored at -80°C.

Total RNA was isolated using RNAqueous® Total RNA Isolation Kit (Ambion, Waltham, MA, USA) according to the manufacturer’s instructions and treated twice with DNase (Ambion). Subsequently RNA samples were purified by Agencourt RNAClean XP kit (Beckman Coulter, Brea, CA, USA). cDNA was obtained from the RNA using the RevertAid^TM^ H Minus M-MuLV reverse transcriptase and oligo-dT mixture (Fermentas, Vilnius, Lithuania). Primers for *CKX* and *ACT* genes were designed using Primer Express 3.0 software (Applied Biosystems, Foster City, CA, USA). The real-time reaction mixtures contained diluted cDNA samples, POWER SYBR Green PCR Master Mix and 300 nM of each primer. All cDNA samples were run in four technical replications on the StepOne-Plus Real-Time PCR System in a default program (Applied Biosystems). Cycle threshold values were normalized with respect to the actin gene.

### Site-directed mutagenesis

Primers for site-directed mutagenesis were designed using web-based QuikChange Primer Design program and the reaction was carried with QuikChange II XL Site-Directed Mutagenesis Kit (Agilent Technologies, Santa Clara, CA, USA) according to the instruction manual. Mutations were introduced into the *NoCKX1* gene in pDrive vector (Qiagen). The Y68 residue was replaced by His (Y68H), L127 residue by Asp (L127D) and Y427 residue by Phe (Y426F) in several combinations as single, double or triple mutations. Correct generation of desired mutations was confirmed by DNA sequencing and respective genes were subcloned into pCIOX vector for protein expression and purification.

### Identification of recombinant proteins by MALDI-TOF mass spectrometry

Protein bands (containing picomolar protein amounts) were excised from Coomassie-stained SDS-PAGE gels and mass analysis was performed as previously described [37] with Microflex LRF20 MALDI-TOF mass spectrometer (Bruker Daltonik, Bremen, Germany) equipped with a microScout ion source and a 337-nm nitrogen laser (10 Hz).

### IPT activity assay

Routine activity assay was performed at 25°C overnight in 200 μl of a reaction mixture consisting of 100 mM Tris/HCl buffer, pH 7.5, containing 10 mM MgCl_2_, with 100 μM AMP and 100 μM DMAPP (Echelon BioSciences, Salt Lake City, UT, USA) as substrates and 100 μl of purified enzyme. To assess substrate preference of NoIPT1, ADP or ATP were used as isoprene chain accepting substrates, whereas isopenthenyl diphosphate or HMBPP (both from Echelon Bioscences) as isoprene chain donating substrates. The reaction was initiated by adding the isoprenoid substrate and stopped by heating to 95°C for 5 min to inactivate the enzyme. For determination of Michaelis-Menten constants, the reaction mixtures containing a suitable amount of enzyme and varying substrate concentrations were incubated for 1 to 4 h. The *K*_m_ for DMAPP was determined at fixed AMP concentration of 20 μM, while *K*_m_ for AMP was determined at DMAPP concentration of 150 μM. Reactions were conducted in duplicate and each constant was determined in two independent assays. The tRNA IPT activity of NoIPT1 was tested using DMAPP and a synthetic 17-base consensus oligoribonucleotide with a sequence based on the unmodified stem-loop region of tRNA^Phe^ [44] as substrates followed by hydrolysis with nuclease P1 and dephosphorylation by alkaline phosphatase [45].

The IPT activity assay was based on determination of reaction products by HPLC or capillary electrophoresis with UV detection at 268 nm. Cytokinin ribosides and corresponding monophosphates were determined on Symmetry C18 column (2.1 x 150 mm, 5 μm; Waters, Milford, MA, USA) connected to Alliance 2695 high performance liquid chromatograph (Waters). The column was eluted by a linear gradient of 15 mM ammonium formate, pH 4.0 (A) and methanol (B) using the following solvent mixture: 0–25 min, 5–60% B, 25–26 min, 60–100% B, 26–27 min 100% B. Linear gradient of 15 mM ammonium formate, pH 4.0 (A) and acetonitrile (B) was used for analysis of oligoribonucleotide hydrolysates (0–30 min, 5–24% B, 30–31 min, 24–100% B). The flow rate was 0.25 ml/min and the column temperature was 30°C. The concentration of product was determined by a calibration curve method using authentic standard compounds (Olchemim, Olomouc, Czech Republic) [46,47]. Capillary electrophoresis determination of cytokinin di- and triphosphates was performed as described [48].

### CKX activity assay

Cytokinin dehydrogenase activity was measured using a modified end-point method described earlier [49]. The reaction mixture (total volume of 0.6 ml in 1.5 ml tube) consisted of 100 mM McIlvaine buffer, pH 5.0 or 7.5, electron acceptor, substrate (dissolved in dimethyl sulfoxide that reached the final concentration of 2.5% in the reaction mixture), and an appropriate concentration of the enzyme sample. As electron acceptors 500 μM 2,3-dimetoxy-5-methyl-1,4-benzoquinone (Sigma-Aldrich) and 2,6-dichlorophenol indophenol (LOBA Feinchemie, Fischamend, Austria) were used in pH 5.0 and 7.5, respectively, while ferricyanide (Lachema, Brno, Czech Republic) was used in both pH conditions. As substrates 250 μM *N* ^*6*^-(2-isopentenyl) adenine, *N* ^*6*^-(2-isopentenyl) adenine 9-riboside, *N* ^*6*^-(2-isopentenyl) adenine 9-glucoside and 6-benzyladenine were used. After addition of substrate, the reaction mixtures were incubated for 18 h at 37°C. The enzymatic reaction was stopped by the addition of 0.3 ml of 40% trichloroacetic acid and subsequently 0.2 ml of 4-aminophenol (2% solution in 6% trichloroacetic acid) followed by samples centrifugation at 19,500 *g* for 5 min to remove protein precipitate. In order to determine the concentration of product Schiff base specific for a given substrate the absorption spectrum in the range of 300–500 was recorded by Agilent 8345 diode array spectrophotometer (Agilent Technologies, Santa Clara, CA, USA).

### Phylogenetic analysis

In order to infer the phylogenetic relationships of IPTs and CKX proteins in cyanobacteria and plants, homologous sequences in the predicted proteomes of sixteen cyanobacteria (eleven species), the plant pathogen *R*. *fascians* and nine plant species were identified (S2 Table). Sequences were obtained from the following sources: cyanobacterial sequences from Uniprot (http://www.uniprot.org/), sequences for *Arabidopsis thaliana*, *Oryza sativa*, *Vitis vinifera*, *P*. *patens*, *Selaginella moellendorffii*, *Chlamydomonas reinhardtii* and *Ostreococcus lucimarinus* from Phytozome (v9.1) (http://www.phytozome.net), *Picea abies* from spruce genome project database (http://congenie.org/start), *Amborella trichopoda* from the Amborella genome database (www.amborella.org) and for *R*. *fascians* from GenBank (http://dx.doi.org/10.7267/N9PN93H8), similar to a previously described approach [50]. Briefly, the predicted proteomes were searched for homologs by identifying protein domains using HMMER3 (hmmsearch, gathering cutoff; CKX domain (PF09265); IPT domain (PF01745); IPPT domain (PF01715)) [51]. For the CKX homologs, proteins domains were extracted according to the HMMER3 results. Proteins sequences were aligned (in the case of the IPT/IPPT after merging the results) using MAFFT (v7.047) [52], and alignments were subsequently manually assessed to remove short fragments and proteins derived from gene isoforms. To further remove poorly aligned positions in the alignments, the heuristic approach of trimAl [53] was used to trim the alignment. Subsequently, phylogenetic trees were constructed using RAxML (v8.0.5) [54] under the fast optimization method and searched for the best scoring maximum-likelihood (ML) tree using the amino acid substitution matrix Whelan and Goldman (WAG) with gamma model for rate of heterogeneity; the robustness of the inferred tree topology was assessed with 500 bootstrap replicates.

## Results

**Fig 1:**
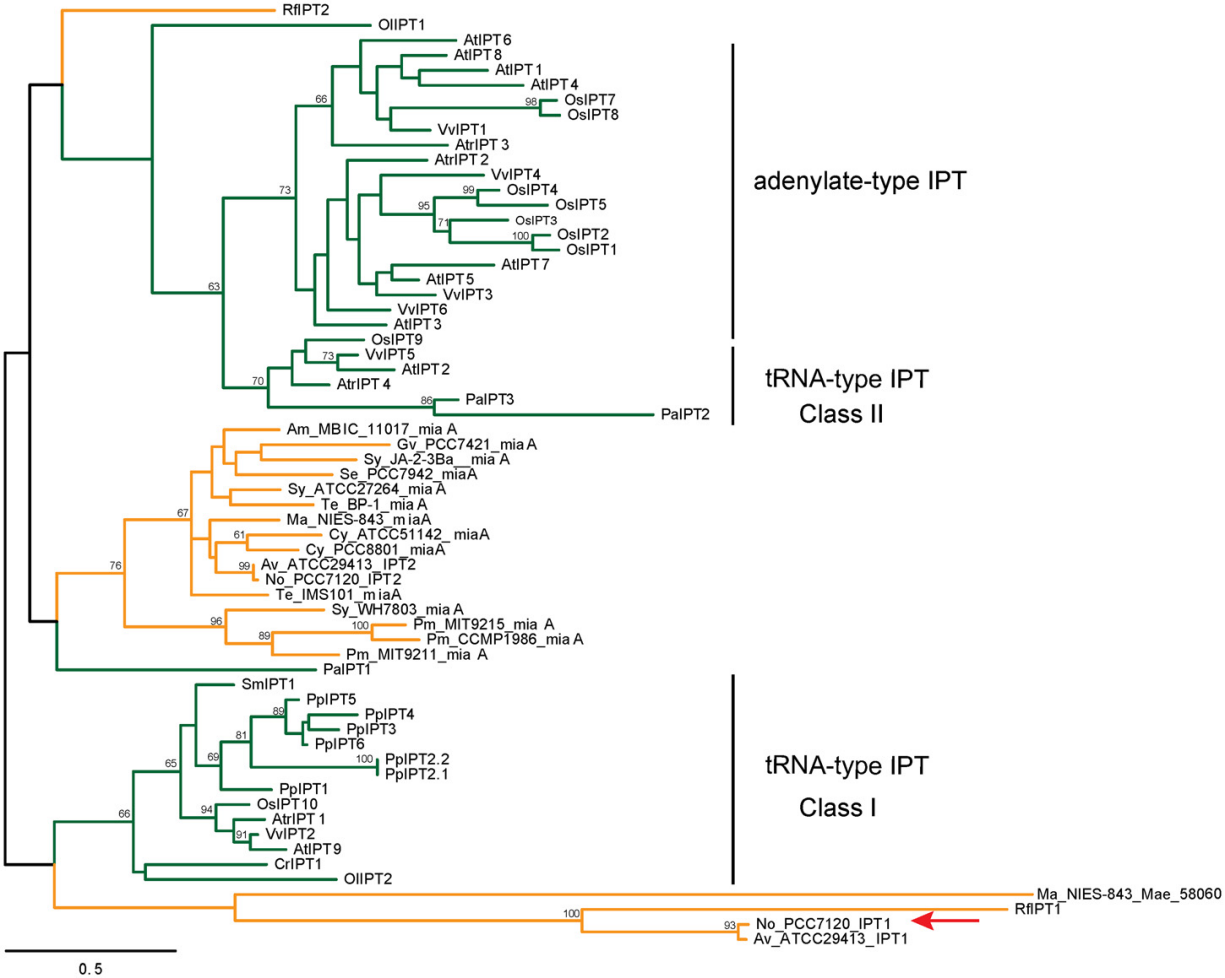
Phylogenetic tree for the IPT proteins shows diverse pattern for the different subtypes. Maximum likelihood phylogeny derived by the full-protein sequences identified in both plants (green) and bacteria (orange). The robustness of the phylogenetic tree was assessed using 500 bootstrap repetitions. The sequence identifiers and species corresponding to the abbreviations are listed in S2 Table. The investigated sequence is marked with a red arrow. The tRNA IPTs clades were named as suggested before [9].

Previously, a gene (PCC7120DELTA_RS03075, named NoIPT1) which resembles bacterial and plant adenylate IPT genes, has been reported in the cyanobacterium *Nostoc* sp. PCC 7120 [19]. The predicted NoIPT1 protein shares 15 to 22% identity with IPT proteins from plants and phytopathogenic bacterium *R*. *fascians* (S2 Table). A single sequence with similarity to CKX encoding gene had been also found [29]. NoCKX1 protein shows low identity with plant CKX proteins (between 17 and 26%) as well as with CKX from *R*. *fascians* (27%) (S2 Table). To understand the evolutionary history of these proteins, a stringent analysis was conducted using Hidden Markov Model search with a large dataset containing nine plant species, eleven cyanobacterial species and the plant pathogen, *R*. *fascians*, for which IPT and CKX genes have been reported earlier [2] (S2 Table, S1 and S2 Figs). As shown in Fig 1, two cyanobacterial IPTs (NoIPT1 and AvIPT1) clustered with RfIPT1, all proteins being predicted as adenylate IPTs [2,9]. The next closely related protein was from the cyanobacterial species, *Microcystis aeruginosa*. The most closely related plant IPTs were tRNA IPTs of the class I (prokaryotic-type tRNA IPTs), which included all IPTs from *P*. *patens*, and also IPT9 from Arabidopsis and IPT10 from rice. The other IPT sequences from both, bacteria and plants, were clearly distinct. The remaining cyanobacterial IPTs formed a clade, which was clearly separated from both, the plant tRNA IPTs and adenylate IPTs (Fig 1). While the bootstrap values are generally rather low, the overall architecture of the IPT tree is in parts similar to what has been published previously using a different bioinformatic approach [9].

**Fig 2:**
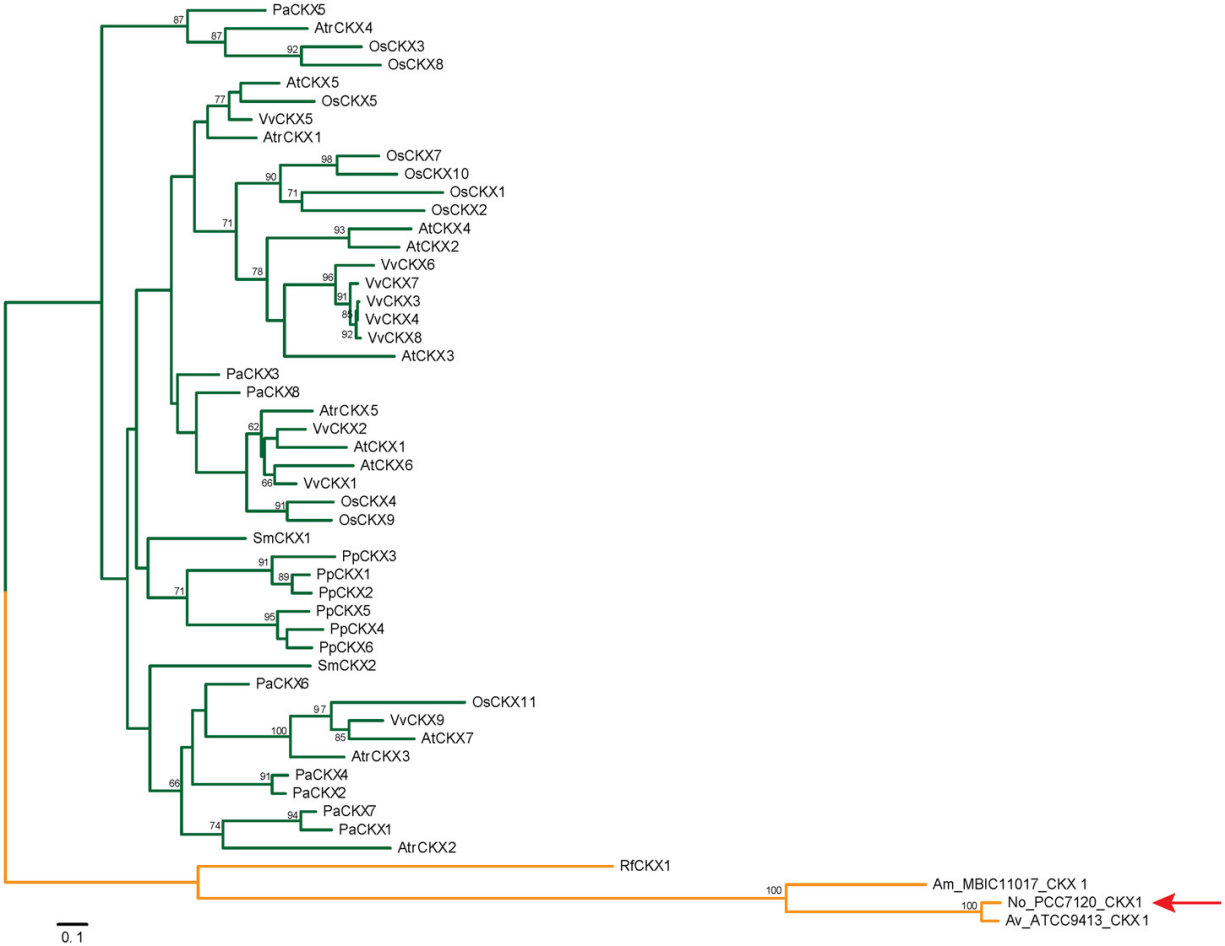
Phylogenetic tree for the CKX proteins shows a clear distinction between plants and bacterial sequences. Maximum likelihood phylogeny derived by the CKX domain sequences identified in both plants (green) and bacteria (orange). The robustness of the phylogenetic tree was assessed using 500 bootstrap repetitions. The sequence identifiers and species corresponding to the abbreviations are listed in S2 Table. The investigated sequence is marked by a red arrow.

For the CKX proteins only four bacterial sequences, one in each bacterium, were detected, all of which clearly clustered separately from the plant sequences. The three cyanobacterial proteins, NoCKX1, AvCKX1, and AmCKX1, were closely related, while the fourth, RfCKX1 from the pathogen *R*. *fascians*, was separated from these three CKXs by a long branch (Fig 2). However, all four sequences are similar to each other and clearly separated from the plant CKXs. Thus the phylogenetic tree for the CKX was similar to that reported previously [2].

### *Nostoc* sp. PCC 7120 produces a spectrum of cytokinins

To provide supporting evidence for the presence of the cytokinin metabolic apparatus in *Nostoc*, cytokinins were analyzed in *Nostoc* cells, using UPLC/MS. With the exception of cytokinin *N*9-glucosides, the cyanobacterium contained all common metabolites of isoprenoid cytokinins, including free bases, ribosides, nucleotides and *O*-glucosides, the most abundant being *cis*-zeatin derivatives (Table 2). Cytokinin nucleotides were generally the most abundant metabolites, followed by free bases and ribosides. The levels of *O*-glucosides were in most cases significantly lower than those of respective zeatin derivatives (Table 2).

**Table 2:** Concentration of cytokinins in *Nostoc* cells.

Cytokinin	Cytokinin concentration (pmol/g FW)
iP^a^	13.09 ± 1.43
iPR	6.66 ± 1.39
iPNT	14.74 ± 2.47
**Total iP derivatives**	**34.49**
tZ	0.23 ± 0.18
tZR	0.03 ± 0.01
tZNT	0.30 ± 0.16
tZOG	0.09 ± 0.03
tZROG	0.003 ± 0.00
**Total tZ derivatives**	**0.653**
cZ	11.37 ± 1.84
cZR	7.57 ± 4.53
cZNT	33.59 ± 11.46
cZOG	1.72 ± 0.16
cZROG	0.22 ± 0.06
**Total cZ derivatives**	**54.47**
DHZ	0.43 ±0.18
DHZR	0.16 ± 0.04
DHZNT	2.97 ± 1.82
DHZOG	0.07
DHZROG	0.02 ± 0.00
**Total DHZ derivatives**	**3.65**

^a^Abbreviations: iP, *N* ^*6*^- (Δ^2^-isopentenyl) adenine; iPR, *N* ^*6*^- (Δ^2^-isopentenyl) adenine 9-riboside; iPNT, *N* ^*6*^- (Δ^2^-isopentenyl)adenine nucleotides; tZ, *trans*-zeatin; tZR, *trans*-zeatin 9-riboside; tZNT, *tran*s-zeatin nucleotides; tZOG, *trans*-zeatin-O-glucoside; tZROG, *trans*-zeatin 9-riboside-O-glucoside; cZ, *cis*-zeatin; cZR, *cis*-zeatin 9-riboside; cZNT, *ci*s-zeatin nucleotides; cZOG, *cis*-zeatin-O-glucoside; cZROG, *cis*-zeatin 9-riboside-O-glucoside; DHZ, dihydrozeatin; DHZR, dihydrozeatin 9-riboside; DHZNT, dihydrozeatin nucleotides; DHZOG, dihydrozeatin-O-glucoside; DHZROG, dihydrozeatin 9-riboside-O-glucoside.

### *NoIPT1* gene encodes functional adenylate isopentenyl transferase

**Fig 3:**
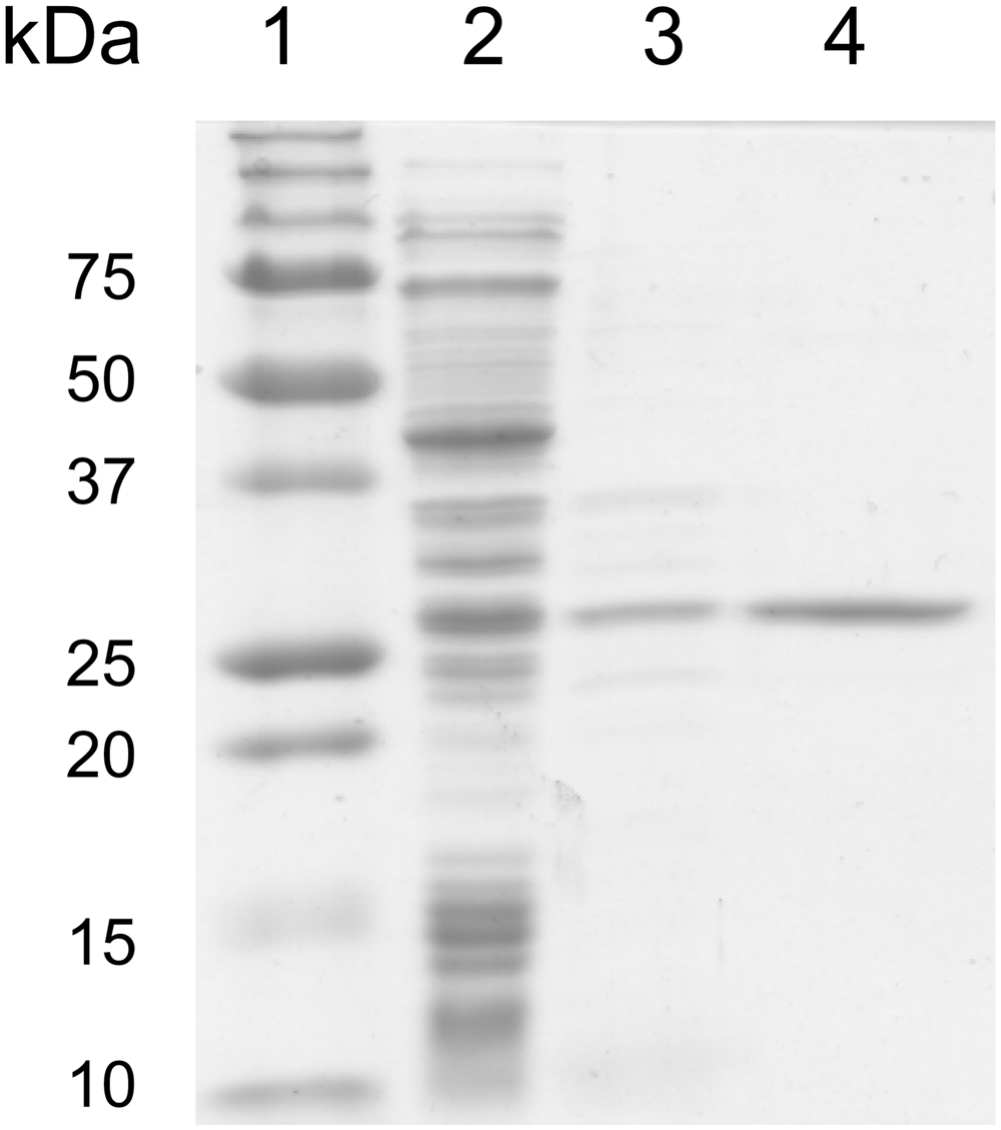
SDS-PAGE analysis of the purified *NoIPT1*. Lane 1: molecular mass standards; lane 2: cell lysate from *E*. *coli* induced by 0.5 mM IPTG; lane 3: fraction collected from High Q column; lane 4: *NoIPT1* purified by Ni-NTA affinity chromatography. Proteins were separated in 12.5% SDS-polyacrylamide gel.

To confirm the ability of NoIPT1 to synthesize cytokinins, the protein was heterologously expressed in *Escherichia coli* with an added N-terminal polyhistidine tag to facilitate purification from cell lysates. Despite extensive optimization of the purification procedure, one-step affinity purification on Ni-NTA matrix was not sufficient to obtain homogenous protein and thus the ion-exchange chromatography on High Q was performed prior to loading the protein extract onto Ni-NTA column. The purified recombinant protein was examined by SDS-PAGE to estimate molecular mass and to evaluate the effectivity of the purification process (Fig 3). The observed molecular mass of NoIPT1 was 27.9 ± 0.42 (SD) kDa, which is consistent with the theoretical molecular mass of 28.3 kDa.

To characterize biochemical properties of NoIPT1, its activity was examined with DMAPP and AMP as substrates. The optimum pH for the reaction was found to be at pH 7.5. The specific activity of purified enzyme under these conditions was 187 pmol/s mg. The *K*_m_ values for AMP and DMAPP were estimated to be 0.63 μM and 27.05 μM, respectively (standard deviations were 0.18 and 2.47 μM, respectively). ATP and ADP appeared not to be substrates of NoIPT1, since the formation of *N* ^*6*^-isopentenyladenosine -5´- triphosphate or *N* ^*6*^-isopentenyladenosine -5´- diphosphate was never detected. However, a small amount of *N* ^*6*^- isopentenyladenosine -5´- monophosphate (iPMP) was formed and, at the same time, partial conversion of ATP to ADP and AMP or ADP to AMP could be observed. Although the protein was purified to apparent homogeneity (Fig 3), it appears that it contained a contaminating phosphatase enzyme presumably originating from bacterial cells. To exclude that IPT itself possess phosphatase activity, a control experiment with cells transformed using empty pET-28b(+) vector was performed. Although no protein was detected in a fraction eluted from Ni-NTA column either by Bradford method or on SDS-PAGE gel, ATP was converted to ADP and AMP upon incubation with this extract, indicating that the NoIPT1 was indeed contaminated by trace amount of bacterial phosphatase(s). The possibility that iPMP formed in the reaction of enzyme with ATP/ADP and DMAPP originated from de-phosphorylation of *N* ^*6*^-isopentenyladenosine -5´- diphosphate or *N* ^*6*^-isopentenyladenosine -5´- triphosphate can be ruled out by comparing ATP, ADP and AMP concentrations in reaction mixtures with and without DMAPP: the concentrations of ATP and ADP in both reaction mixtures were same, while AMP concentration was lower in the presence of DMAPP, since they reacted to form iPMP. Other compounds that could be used as side chain donor were also tested in the IPT activity assay in combination with AMP, ADP or ATP. NoIPT1 used a hydroxylated side chain precursor, HMBPP, to form *trans*-zeatin-5´-monophosphate in the reaction with AMP. The reaction rate was however estimated to be only about 0.5% of that with DMAPP (at 100 μM concentration). Isopentenyl diphosphate, an isomer of DMAPP, was not substrate with either of the side chain acceptor substrates. NoIPT1 did not show tRNA IPT activity, as it did not prenylate synthetic tRNA-stem-loop minihelix substrate.

### Production of recombinant *NoCKX1*

**Fig 4:**
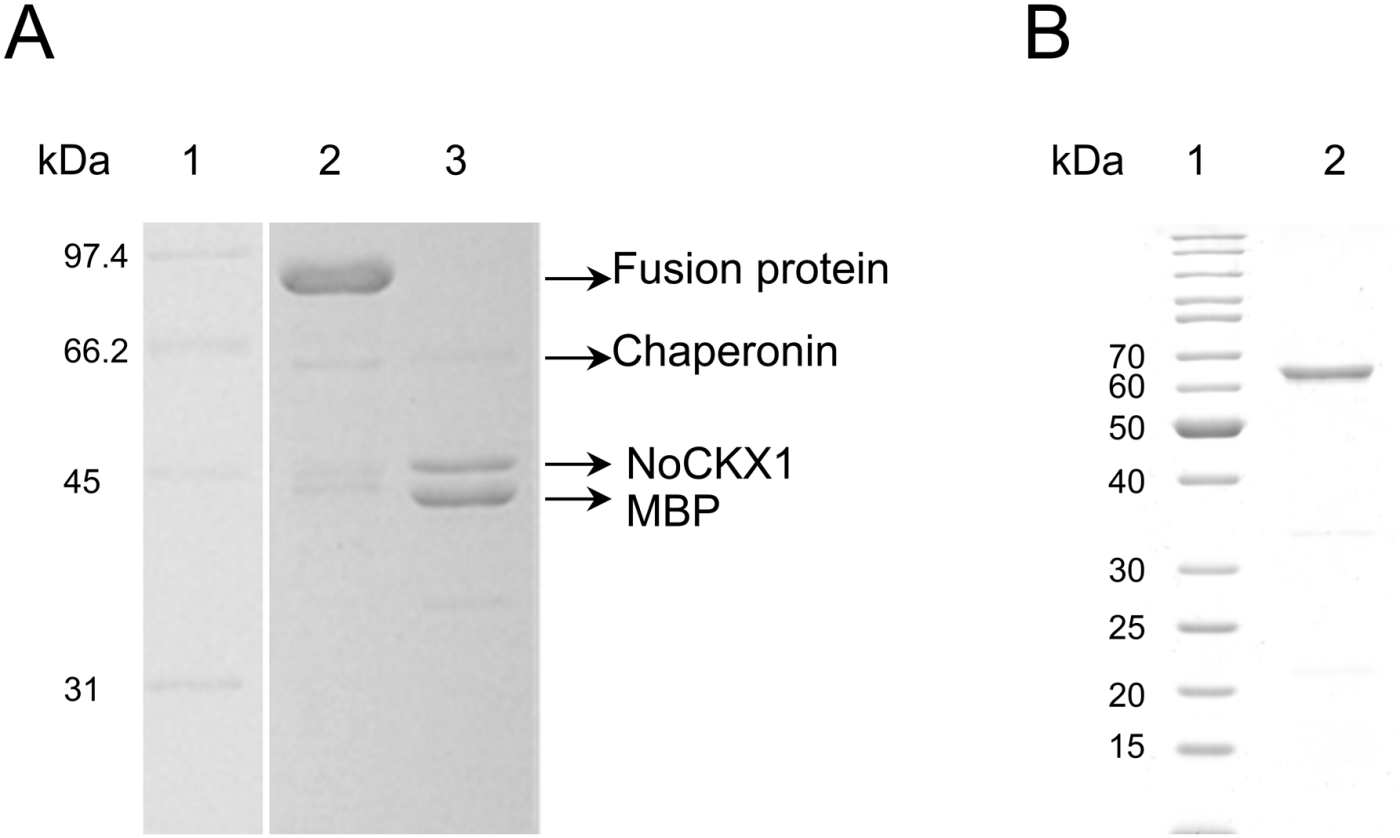
SDS-PAGE analysis of purification of *NoCKX1* expressed from different vectors. A/ Purification of *NoCKX1* expressed from pMAL-c4X vector. Lane 1: molecular mass standard; lane 2: fusion protein (*NoCKX1* with maltose binding protein) after purification on amylose column; lane 3: same protein after cleavage by factor Xa protease. B/ Purification of *NoCKX1* expressed from pCIOX vector. Lane 1: molecular mass standard; lane 2: fusion protein (*NoCKX1* with SUMO and histidine tag) after purification on Ni-NTA column. Proteins were separated in 10% SDS-polyacrylamide gel.

To see whether *NoCKX1* encodes functional enzyme exhibiting cytokinin degradation activity, a recombinant protein was prepared by means of intracellular heterologous expression in *E*. *coli*. The gene was originally expressed from pQE40 vector, which enables protein purification via histidine fusion tag, but protein expression in this system was very weak. The protein was then successfully expressed from pMAL-c4X vector as a MBP-NoCKX1 fusion protein of about 92 kDa (42.5 kDa MBP and NoCKX1 49.8 kDa). However, CKX activity in clarified cell lysates was not detected. The protein was subsequently purified on amylose resin where most of the ballast proteins were removed, and MBP-NoCKX1 fusion protein was cleaved with Factor Xa protease. The efficiency of MBP cleavage and protein purity was examined by SDS-PAGE that revealed two sharp bands of molecular mass of about 50 and 42 kDa (NoCKX1 and MBP, respectively) and a weaker band of 60 kDa (Fig 4A). The 60 kDa band was subjected to MALDI-TOF analysis that identified presence of *E*. *coli* chaperonin GroEL. MALDI-TOF analysis also confirmed that 50 kDa band belongs to CKX from *Nostoc* sp. PCC 7120. MBP was effectively removed from the protein mixture after repeated loading on amylose resin. The CKX activity of the purified protein was examined using various combinations of cytokinin substrates with electron acceptors at slightly acidic as well as neutral pH to reflect diverse substrate preferences of different CKX proteins [28,37], but formation of expected product was not detected. NoCKX1 was also alternatively prepared using pTYB12 vector which provides chitin binding affinity tag and self-cleavable intein that allows single step purification without the use of a protease and which was successfully used many times in our laboratory to express a number of various plant CKX proteins [28,37]. However, in the case of NoCKX1 the presence of chaperonin and no CKX activity was detected after protein purification. Since the presence of co-purifying chaperonin may indicate protein-folding difficulties, which may be overcome by slowing down the protein expression and/or aiding the folding by co-expression of chaperonins [39], the Arctic Express (DE3) host cells, expressing chaperonins active at low temperatures, were also tested for NoCKX1 production. However, very high ratio of chaperonin to NoCKX1 (up to 90% of chaperonin) was obtained with Arctic Express (DE3) cells for both, pMAL-c4X and pTYB12, vectors.

Finally, the NoCKX1 protein was expressed from pCIOX vector that bears two tags, SUMO (small ubiquitin-like modifier) that stabilizes expressed proteins and 8xHis for easier purification. NoCKX1 purified on Ni-NTA resin was subjected to SDS-PAGE that revealed the presence of a band corresponding to molecular mass of approximately 62 kDa, and two bands with low intensity corresponding to 33 kDa and 22 kDa, respectively (Fig 4B). Subsequent treatment with SUMO protease resulted in protein cleavage to SUMO tag and NoCKX1. CKX activity assays were performed with various substrates and electron acceptors but no activity was found either with SUMO-fused NoCKX1 or the free NoCKX1.

In an effort to remove chaperonin from the recombinant protein, several techniques were applied. First, the protein lysate was incubated with 10 mM MgCl_2_, 5 mM ATP and 150 mM KCl before loading on the column. That should support disintegration of the chaperonin subunit and may stimulate ATPase activity of some chaperonins and therefore lead to release of a protein from chaperonin [39]. Unfortunately, as soon as chaperonin was removed the NoCKX1 protein degraded. The same happened when the lysate incubated with ATP, MgCl_2_ and KCl was treated by 2.3 M urea and, after another incubation and dialysis, purified on column using elution with 2.3 M urea [40]. The last method used to separate chaperonin was applied on purified NoCKX1. The protein was dialysed overnight against 8 M urea, 6 M guanidine-HCl or 1–4% Chaps, respectively, followed by FPLC purification on Superdex, but the separation was not successful.

The lack of activity of NoCKX1 expressed in any of expression systems probably relates to the fact that the purified protein does not contain FAD cofactor: characteristic yellow color and absorption spectrum of flavoproteins was not observed. Cultivation of bacteria and purification of NoCKX1 in the presence of FAD did not result in insertion of FAD cofactor. Production of recombinant NoCKX1 in *Pichia pastoris* expression system either intracellular or extracellular was not achieved. Protein expression was never confirmed and no CKX activity was detected. The attempts to detect intrinsic CKX activity directly in *Nostoc* cell extract (produced from cells cultivated for 16 or 21 days under the conditions described in ´Cytokinin analysis´) were not successful as well.

### Site-directed mutagenesis of *NoCKX1* gene

**Fig 5:**
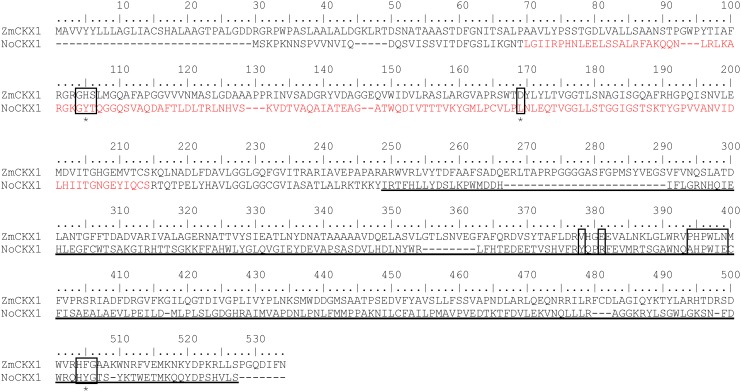
Amino acid sequence alignment of ZmCKX1 and *NoCKX1*. Protein sequences were aligned using the ClustalW interface in BioEdit 7.0.5.3 [55]. Major differences in conserved regions are indicated by boxes. Amino acid residues which were mutated to match consensus sequence are marked by asterisk. Histidine in the motif ^104^GHS is involved in FAD covalent linkage, ^394^PHPWLN and ^504^HFG are conserved C-terminal domains and the residues ^169^D, ^378^V and ^381^E are involved in substrate biding. FAD binding domain (pfam01565) and cytokinin binding domain (pfam09265) of *NoCKX1* identified by NCBI´s conserved domain search [66] are shown in red and underlined letters, respectively. Numbering refers to the sequence of ZmCKX1.

The lack of CKX activity in the prepared recombinant NoCKX1 proteins may be the consequence of substantial number of amino acid substitutions in the cyanobacterial CKX sequence, including the conserved domains, when compared to plant CKXs (see Fig 5 for sequence alignment created in BioEdit [55]). These include three domains: (i) the GHS domain, where a conserved histidine residue participating in FAD cofactor binding [56] is replaced by tyrosine in NoCKX1, (ii) an aspartate residue facilitating oxidation of cytokinin substrate [56], which is replaced by leucine, and (iii) the HFG domain, where phenylalanine residue crucial for protein activity and stability [28] is replaced by tyrosine. Together six different mutant variants of NoCKX1 were prepared: single mutants Y68H, L127D, and Y426F, double mutants Y68H L127D and L127D Y426F, and triple mutant Y68H L127D Y426F. The expression of mutated NoCKX1 proteins was carried in pCIOX vector. Proteins were purified from clarified cell lysates by affinity chromatography on Ni Sepharose and assayed for CKX activity. However, none of the mutants showed CKX activity. In addition, none of the mutant proteins contained the FAD cofactor.

### Transgenic tobacco plants

Since overexpression of CKX in tobacco can result in a strong cytokinin-deficient phenotype [57,58], transgenic tobacco plants with CKX from *Nostoc* sp. PCC 7120 were prepared to further test the functionality of *NoCKX1*. *NoCKX1* was cloned and positioned under the control of a constitutive 35S promoter. In addition, NoCKX1 targeted to cytosol was given an apoplastic signal sequence from AtCKX2 and also a vacuolar signal sequence from AtCKX1. Therefore, three constructs were used for further transformation of tobacco plants. At least 15 independent transformants were obtained for each of the three genes. Total RNA extraction from transgenic tobacco leaves was carried out to confirm the expression of the genes of interest. A qPCR experiment was designed with tobacco *act* gene as an endogenous control and WT *Nicotiana tabacum* as a reference. Cycle threshold values were normalized with respect to the actin gene. All plants were shown to be transgenic. The expression level of target genes was increased at least 50 times up to even 10,000 times. However no plant transformed with *NoCKX1* demonstrated the elevated CKX activity or any phenotypic change in the aerial part and the root system. Also T1 and T2 generation of these transgenic plants did not show phenotypic changes even though the expression level of target genes was increased compared to wild type.

## Discussion

Cyanobacteria are able to synthesize various classes of plant hormones, such as auxins, cytokinins, gibberellins, abscisic acid, ethylene, and jasmonates [59,60]. Therefore, it is not surprising that genes homologous to *Arabidopsis* IPTs are also found in cyanobacteria, e.g. *Nostoc* sp. PCC 7120 contains one gene homologous to adenylate IPTs (NoIPT1) and one gene annotated as tRNA IPT (NoIPT2) [2,19]. However, the majority of cyanobacteria only encode tRNA IPTs, whereas cyanobacterial adenylate IPTs are scarce. Interestingly these adenylate IPTs of cyanobacteria are more closely related to plant tRNA IPTs than to the plant adenylate IPTs (Fig 1). This indicates that while they are functionally similar, their biochemical properties might have developed independently of each other, as no IPTs with annotated adenylate IPT activity are found in algae or early diverging land plants, such as *P*. *patens* or *S*. *moellendorffii*. This observation and the structure of the phylogenetic tree presented indicate that tRNA- and adenylate-type IPTs have developed several times independently of each other in different species. This hypothesis is further supported by the fact that also the plant tRNA IPTs branch into two clearly distinguishable clades: tRNA IPT class I, which contains sequences from all land plants and is most closely related to the cyanobacterial adenylate IPTs, and tRNA IPT class II, which is very closely related to the adenylate IPTs from modern land plants, but not from early diverging land plants, such as *P*. *patens* and *S*. *moellendorffii*. The IPTs from cyanobacteria also fall into two clearly separated groups. The first group contains NoIPT1 investigated in present study and RfIPT1 [2], which were both biochemically characterized as functional adenylate IPTs. The second group comprises all remaining cyanobacterial IPTs (including the second IPT from *Nostoc* sp. PCC 7120, NoIPT2), which are quite distinct from all groups of plant IPTs. It is impossible to reliably predict the type of IPT solely based on its sequence and thus experimental studies of the protein function are absolutely necessary. This was clearly demonstrated in a study showing that despite all amino acid sequences of IPTs present in *Physcomitrella* look like tRNA IPT of the class I, cytokinin analysis in *Ppipt1* mutants suggested the presence of a tRNA-independent cytokinin biosynthesis pathway [9]. Whether one or more tRNA IPTs of *Physcomitrella* acquired adenylate IPT function remains to be determined.

The studies published so far on adenylate IPTs from various organisms indicate that bacterial IPTs generally use AMP as prenyl chain acceptor and both DMAPP and HMBPP as prenyl chain donors, while plant IPTs only use DMAPP to prenylate preferentially ATP or ADP [61]. *Nostoc* IPT only uses AMP as side chain acceptor, similar to *Agrobacterium tumefaciens* Tzs and Tmr proteins [61]. On the other hand, it resembles IPTs of plant origin in the reactivity with the side chain donors, as the activity with HMBPP is marginal. Observed reactivity with substrates corresponds well with the absence of amino acid residues in the amino acid sequence of NoIPT1that have been identified as critical to substrate specificity for both prenyl donor and prenyl acceptor, respectively [62,63]. Two positively charged lysine residues (K220, K275) responsible for binding of β- and γ-phosphate groups of ATP in hop IPT [63] are replaced by hydrophobic residues T171 and A223 in NoIPT1. The third lysine residue of hop IPT interacting with β- and γ-phosphate groups, K63, is in the NoIPT1 replaced by corresponding R34, which was shown to form a hydrogen bond with α-phosphate group of AMP in the *Agrobacterium* IPT, Tzs [62,63]. Concerning the substrate specificity for prenyl donor substrate, H214 and D173 residues forming a hydrophilic cavity for a prenyl group and crucial for the reactivity with HMBPP [62] are both replaced in the *Nostoc* IPT: the corresponding residues are V173 and Y216. NoIPT1 still reacts with HMBPP, but at very low rates that did not allow more detailed biochemical characterization. The low reactivity of the enzyme with HMBPP is also reflected in the cytokinin content in *Nostoc* cells, where concentration of isopentenyladenine derivatives is much higher than that of *trans*-zeatin derivatives (Table 2). NoIPT1 did not exhibit tRNA IPT activity. The result is in agreement with the amino acid sequence of NoIPT1, in which several residues conserved in tRNA IPTs of various organisms and shown to be essential for tRNA substrate binding [44] are absent in NoIPT1. On the other hand, these residues are all present in sequence of NoIPT2, which is annotated as tRNA IPT. While in plants adenylate IPTs are responsible for biosynthesis of isopentenyladenine and *trans*-zeatin type cytokinins and *cis*-zeatin type cytokinins are released during tRNA breakdown [7], cytokinins are only found in the form bound to tRNAs in most bacteria. The isopentenyl modifications of tRNA have a fundamental role in translation efficiency and fidelity and thus affect also metabolic functions (for review see [64]). The isopentenyl-modified nucleotides are located in position 37 of anti-codon loop of tRNAs that read codons starting with U [64]. The first step leading to synthesis of cytokinin derivatives in tRNA is formation of isopentenyladenosine by tRNA IPT, which is followed by methylthiolation and, in some organisms, by *cis*-hydroxylation [64]. The high content of *cis*-zeatin metabolites detected in *Nostoc* cells together with substrate specificity of its adenylate IPT indicate the indirect biosynthesis of *cis*-zeatin derivatives via tRNA degradation in this bacterium, although the possibility of direct biosynthesis from *cis*-HMBPP as prenyl donor or *cis*-hydroxylation of isopentenyladenine cannot be ruled out completely.

Besides cytokinin biosynthetic genes, *Nostoc* sp. PCC 7120 contains a single gene coding for hypothetical CKX protein named here NoCKX1. In contrast to IPT proteins, there were very few bacterial CKX sequences detectable. This might indicate that bacteria metabolize cytokinins differently from plants. The only functionally described CKX protein from bacteria, RfCKX1, originates from a plant pathogen, which uses cytokinins in its strategy to manipulate its plant host [30]. Although the sequence of the NoCKX1 protein has rather low similarity to plant CKX proteins (17 to 26% identity, 35 to 46% similarity, S2 Table) as well as to CKX from *R*. *fascians* (27% identity, 44% similarity), both the cytokinin binding domain and the FAD binding domain are predicted to be present by PFAM [65]. Nevertheless, even highly conserved protein regions display many alterations. For instance, *Nostoc* sp. PCC 7120 contains a GYT motif instead of GHS motif, of which histidine is necessary for the covalent binding of FAD cofactor through the 8-methyl group of the isoalloxazine ring. Other differences in the sequence of NoCKX1 include several substrate-binding residues, the most critical being the substitution of active site aspartate residue for leucine, and C-terminal HFG domain where phenylalanine is replaced by tyrosine (Fig 5). The HFG domain is strongly conserved in plant CKX enzymes and the only known exceptions among monocots are barley HvCKX9 (with HYG) and maize ZmCKX6 (with HLG) where the latter enzyme was shown to be inactive due to this specific mutation [28]. When the NoCKX1 protein was expressed in various *E*. *coli* expression systems, the CKX activity was never detected. In all expression systems, a chaperonin co-purified with NoCKX1 protein. The chaperonin was tightly bound to NoCKX1 and its release from NoCKX1 caused a loss of the target protein. In addition, all NoCKX1 fusion proteins did not contain FAD cofactor, although N-terminal domain (residues 36 to 172) is identified as an FAD binding domain by NCBI´s conserved domain search [66]. Several mutant variants of NoCKX1 aimed on introduction of amino acid residues critical to enzyme function were also all inactive and without FAD. Covalent protein flavinylation is a post-translational and self-catalytic process [67] that requires at least three catalytic amino acid residues facilitating formation of the covalent flavin bond with a suitable ligand residue. However, there is no conserved sequence motif among various covalent flavoproteins that could be used to predict covalent flavin binding. Based on the crystal structure [56], the catalytic residues tyrosine (Y491), aspartate (D169, which is also the active site residue of mature enzyme) and its proximal tyrosine (Y170) may facilitate FAD binding via 8-methyl group to ND1 of histidine (H105) in ZmCKX1 [67]. While the only known functional bacterial CKX from *R*. *fascians* [30] contains all corresponding residues (Y393, D117, Y118 and H60), NoCKX1 contains only the C-terminal tyrosine (Y410). The other two catalytic residues cannot be predicted because the corresponding sequence region in NoCKX1 is largely different from either ZmCKX1 or RfCKX1. Moreover, the histidine ligand is replaced with tyrosine (Y68). Despite several prepared mutants, FAD insertion in NoCKX1 was not achieved.

Tobacco plants transformed with NoCKX1 gene did not show any cytokinin-deficient phenotype and the presence of NoCKX1 protein was never confirmed, although the gene was expressed on RNA level. Moreover, the activity of native NoCKX1 in *Nostoc* cells was not detected. These data together indicate that although the corresponding gene is predicted to contain the basic determinants of a cytokinin dehydrogenase it seems not to encode a functional cytokinin dehydrogenase. Several other cyanobacteria such as *Coleofasciculus chthonoplastes* PCC 7420 and *Nodularia spumigena* CCY 9414, which were not included in present study, were shown to contain genes coding proteins homologous to plant CKX showing similar sequence variability as NoCKX1 [2]. With the ever-growing number of sequenced cyanobacterial genomes, it is not unlikely that new cyanobacterial CKX proteins will be uncovered. However, as we could not detect CKX activity for NoCKX1, it may be possible that these proteins have a different function and metabolize other substrates. After being transferred to plants during endosymbiosis, these proteins might have acquired the function in cytokinin degradation, as we find it today in land plants. In this context it is especially surprising that in contrast to land plants no sequence homologous to CKX has been described in algae so far.

Besides the irreversible side chain cleavage by CKX, formation of biologically inactive *N*9-cytokinin glucosides is considered as another irreversible cytokinin deactivating process in land plants [2]. However, this deactivation pathway can be ruled out in *Nostoc*, since *N*9-cytokinin glucosides were not detected in cyanobacterial cell extract. A possible alternative might be a hydrolytic degradation pathway via adenine/adenosine deaminases, which are common enzymes in many bacteria and often hydrolyze also adenine N6 substituted side chain such as methylamine. Their yeast analogs were shown to hydrolyze cytokinins albeit at low rates when compared to adenine [68]. Highly specific cytokinin deaminase was found in marine agarolytic bacterium *Pseudomonas atlantica* [69], but no homologous gene is found in *Nostoc* sp. PCC 7120.

## Conclusions

This study investigated biochemically two predicted proteins, an IPT and a CKX, from cyanobacterium *Nostoc* sp. PCC 7120. While NoIPT1 proved to be functional, no activity was found for putative CKX. This may indicate that while *Nostoc* sp. PCC 7120 and other cyanobacteria are able to synthesize cytokinins, they may be not able to break them down via the CKX pathway. This situation is reminiscent of Chlorophyceae algae, which encode several IPT genes in their genomes, but lack the CKX genes.

## Supporting Information

S1 Fig IPT sequences used in phylogenetic analysis. List of all IPT sequences used in this analysis in a FASTA format.
(TXT)

S2 Fig CKX sequences used in phylogenetic analysis. List of all CKX sequences used in this analysis in a FASTA format.
(TXT)

S1 Table Sequences of primers used for amplification of *NoIPT1* and *NoCKX1* genes. (DOCX)

S2 Table Sequence identifiers and corresponding species for the sequences identified in the phylogenetic analysis. (DOCX)

## Abbreviations

CKX: cytokinin dehydrogenase
DMAPP: dimethylallyl pyrophosphate
HMBPP: *(E)*-4-hydroxy-3-methyl-but-2-enyl pyrophosphate
iPMP: *N* ^6^- isopentenyladenosine -5´- monophosphate
IPT: isopentenyl transferase
MBP: maltose binding protein
