# Supplementary material for: Biochemical Characterization of Putative Adenylate Dimethylallyltransferase and Cytokinin Dehydrogenase from Nostoc sp. PCC 7120
Source: PLoS One. 2015 Sep 16;10(9):e0138468. doi: 10.1371/journal.pone.0138468 (PMC4574047; doi:10.1371/journal.pone.0138468)
Supplement: S1 Table — (DOCX) [file pone.0138468.s003.docx]

**Table S1. Primers used for amplification of *NoIPT1* and *NoCKX1* genes.**

| Vector | Primer name | Primer sequence |
| --- | --- | --- |
| pET28b(+) | NoIPT1_NdeI_fw | GGAATTCCATATGCGATTGCATATA |
|  | NoIPT1_SalI_rev | ACGCGTCGACTTAAAGCGTCACA |
| pQE40 | NoCKX_BamHI_fw | CGGGATCCATGAGTAAACCAAAAAACAATTCTCCAG |
|  | NoCKX_PstI_rev | AAAACTGCAGCTAGCTCAGAACATGGCTCGG |
| pTYB12 | pTYB_EcoRI_fw | GGAATTCATGAGTAAACCAAAAAACAATTCTC |
|  | NoCKX_PstI_rev | AAAACTGCAGCTAGCTCAGAACATGGCTCGG |
| pGAPZαA(His)_10_ | NoCKX_SacII_fw | TCCCCGCGGGGATGAGTAAACCAAAAAACAAT |
|  | NoCKXHis_rev | GCTCTAGAGTAGCTCAGAACATGGCTCGG |
|  | pBIN_NoCKX_XbaI_rev | GCTCTAGACTAGCTCAGAACATGGCTCGG |
| pPICZA(His)_10_ | NoCKX_NdeI_fw | GTCCATATGAGTAAACCAAAAAACAATTCTC |
|  | pPIC_HisNoCKX_rev | CCGCTCGAGCTAGCTCAGAACATG |
