# Supplementary material for: Biochemical Characterization of Putative Adenylate Dimethylallyltransferase and Cytokinin Dehydrogenase from Nostoc sp. PCC 7120
Source: PLoS One. 2015 Sep 16;10(9):e0138468. doi: 10.1371/journal.pone.0138468 (PMC4574047; doi:10.1371/journal.pone.0138468)
Supplement: S2 Table — (DOCX) [file pone.0138468.s004.docx]

**Table S2. Sequence identifiers and corresponding species for the sequences identified in the phylogenetic analysis.**

| **IPT Sequences** | | | | |
| --- | --- | --- | --- | --- |
| **Name in tree** | **Name in FASTA** | **GenBank identifier** | **Organism name** | **Identity/Similarity to NoIPT1 (%)** |
| Am_MBIC 11017_miaA | B0CG28 | WP_012164789.1 | *Acaryochloris marina (strain MBIC 11017)* | 20.5/35.5 |
| AtIPT1 | AT1G68460.1\|Athaliana | NP_177013.1 | *Arabidopsis thaliana* | 19.2/32.1 |
| AtIPT2 | AT2G27760.1\|Athaliana | NP_565658.1 | *Arabidopsis thaliana* | 16.8/28.9 |
| AtIPT3 | AT3G63110.1\|Athaliana | NP_567138.1 | *Arabidopsis thaliana* | 15.3/32.4 |
| AtIPT4 | AT4G24650.1\|Athaliana | NP_194196.1 | *Arabidopsis thaliana* | 21.9/39.1 |
| AtIPT5 | AT5G19040.1\|Athaliana | NP_197405.1 | *Arabidopsis thaliana* | 17.1/33.5 |
| AtIPT6 | AT1G25410.1\|Athaliana | NP_173912.1 | *Arabidopsis thaliana* | 20.8/33.4 |
| AtIPT7 | AT3G23630.1\|Athaliana | NP_566735.1 | *Arabidopsis thaliana* | 19.7/34.5 |
| AtIPT8 | AT3G19160.1\|Athaliana | NP_188547.1 | *Arabidopsis thaliana* | 18.4/33.7 |
| AtIPT9 | AT5G20040.1\|Athaliana | NP_851043.1 | *Arabidopsis thaliana* | 14.7/28.6 |
| AtrIPT1 | evm_27.model.AmTr_v1.0_scaffold00021.85\|Atrichopoda | XP_006852479.1 | *Amborella trichopoda* | 15.7/25.9 |
| AtrIPT2 | evm_27.model.AmTr_v1.0_scaffold00025.75\|Atrichopoda | XP_006850780.1 | *Amborella trichopoda* | 17.0/34.3 |
| AtrIPT3 | evm_27.model.AmTr_v1.0_scaffold00079.46\|Atrichopoda | XP_006842304.1 | *Amborella trichopoda* | 17.9/33.8 |
| AtrIPT4 | evm_27.model.AmTr_v1.0_scaffold00095.58\|Atrichopoda | XP_006832880.1 | *Amborella trichopoda* | 16.5/28.8 |
| Av_ATCC29413_IPT1 | Q3M9L2\|Avariab | WP_011319467.1 | *Anabaena variabilis (strain ATCC 29413)* | 93.0/95.9 |
| Av_ATCC29413_IPT2 | Q3MA53\|Avariab | WP_011319289. | *Anabaena variabilis (strain ATCC 29413)* | 21.2/41.2 |
| CrIPT1 | Cre17.g717350.t1.2\|Creinhardtii | XP_001703754.1 | *Chlamydomonas reinhardtii* | 12.4/20.9 |
| Cy_ATCC51142_miaA | B1X0D2 | WP_009545682.1 | *Cyanothece sp. (strain ATCC 51142)* | 22.9/38.1 |
| Cy_PCC8801_miaA | B7K612 | WP_015957294.1 | *Cyanothece sp. (strain PCC 8801)* | 19.9/37.0 |
| Gv_PCC7421_miaA | Q7MBC1 | NP_926194.1 | *Gloeobacter violaceus (strain PCC 7421)* | 20.0/35.6 |
| Ma_NIES-843_Mae_58060 | B0JIS0 | WP_012268014.1 | *Microcystis aeruginosa (strain NIES-843)* | 16.5/27.1 |
| Ma_NIES-843_miaA | B0JN42 | WP_012264058.1 | *Microcystis aeruginosa (strain NIES-843)* | 19.6/36.8 |
| No_PCC7120_IPT1 | Q8Z078 | WP_010994397.1 | *Nostoc sp. (strain PCC 7120 / UTEX 2576)* | 100/100 |
| No_PCC7120_IPT2 | Q8YLN2 | WP_010999390.1 | *Nostoc sp. (strain*  *PCC 7120 / UTEX 2576)* | 20.5/39.3 |
| OlIPT1 | 34266\|Olucimarinus | XP_001416677.1 | *Ostreococus lucimarinus* | 14.4/29.2 |
| OlIPT2 | 3723\|Olucimarinus | XP_001418572.1\| | *Ostreococus lucimarinus* | 15.8/33.1 |
| OsIPT1 | LOC_Os03g24440.1\|Osativa | ABF96060.1 | *Oryza sativa* | 15.5/31.8 |
| OsIPT10 | LOC_Os06g51350.1\|Osativa | NP_001058651.2 | *Oryza sativa* | 15.3/31.6 |
| OsIPT2 | LOC_Os03g24240.1\|Osativa | NP_001050141.1 | *Oryza sativa* | 16.8/33.2 |
| OsIPT3 | LOC_Os05g24660.1\|Osativa | BAE47446.1 | *Oryza sativa* | 17.5/37.0 |
| OsIPT4 | LOC_Os03g59570.1\|Osativa | NP_001051662.1 | *Oryza sativa* | 17.0/31.8 |
| OsIPT5 | LOC_Os07g11050.1\|Osativa | BAC84458.1 | *Oryza sativa* | 15.5/30.0 |
| OsIPT7 | LOC_Os05g47840.1\|Osativa | NP_001056250.2 | *Oryza sativa* | 16.3/28.5 |
| OsIPT8 | LOC_Os01g49390.1\|Osativa | BAN84023.1 | *Oryza sativa* | 16.8/29.1 |
| OsIPT9 | LOC_Os01g73760.1\|Osativa | NP_001045520.1 | *Oryza sativa* | 14.9/28.3 |
| PaIPT1 | MA_158851g0020\|Pabies | WP_024920335.1 | *Picea abies* | 17.9/33.3 |
| PaIPT2 | MA_20274g0010\|Pabies | ABR17233.1 | *Picea abies* | 16.1/29.7 |
| PaIPT3 | MA_54217g0010\|Pabies |  | *Picea abies* | 15.8/31.3 |
| Pm_CCMP1986_miaA | Q7TU25 | WP_011133262.1 | *Prochlorococcus marinus subsp. pastoris (strain CCMP1986 / MED4)* | 18.5/40.1 |
| Pm_MIT9211_miaA | A9BD80 | WP_012196313.1 | *Prochlorococcus marinus (strain MIT 9211)* | 19.3/37.0 |
| Pm_MIT9215_miaA | A8G7E1 | WP_012008515.1 | *Prochlorococcus marinus (strain MIT 9215)* | 18.9/40.2 |
| PpIPT1 | Pp1s96_115V6.1\|Ppatens_v1.6 | ABP88738.1 | *Physcomitrella patens* | 12.9/25.9 |
| PpIPT2.1 | Pp1s137_3V6.1\|Ppatens_v1.6 | XP_001771357.1 | *Physcomitrella patens* | 12.6/22.0 |
| PpIPT2.2 | Pp1s137_19V6.1\|Ppatens_v1.6 | XP_001771407.1 | *Physcomitrella patens* | 14.1/25.7 |
| PpIPT3 | Pp1s280_8V6.1\|Ppatens_v1.6 | XP_001780492.1 | *Physcomitrella patens* | 15.6/26.0 |
| PpIPT4 | Pp1s64_135V6.1\|Ppatens_v1.6 | AGC04673.1 | *Physcomitrella patens* | 13.2/25.8 |
| PpIPT5 | Pp1s14_391V6.1\|Ppatens_v1.6 | AGC04674.1 | *Physcomitrella patens* | 15.2/24.0 |
| PpIPT6 | Pp1s341_1V6.1\|Ppatens_v1.6 | XP_001782787.1 | *Physcomitrella patens* | 14.4/27.2 |
| RfIPT1 | RFD188_04924\|Rfascians | WP_015586134.1 | *Rhodococcus fascians* | 22.1/43.9 |
| RfIPT2 | RFD188_02204\|Rfascians | WP_032383562.1 | *Rhodococcus fascians* | 17.7/38.6 |
| Se_PCC7942_miaA | Q8GIT6 | WP_011378489.1 | *Synechococcus elongatus (strain PCC 7942)* | 18.5/38.8 |
| SmIPT1 | 410898\|Smoellendorffii | XP_002970127.1 | *Selaginella moellendorffii* | 16.6/29.5 |
| Sy_ATCC27264_miaA | B1XKE8 | WP_012306817.1 | *Synechococcus sp. (strain ATCC 27264 / PCC 7002 / PR-6) (Agmenellum quadruplicatum)* | 19.9/35.2 |
| Sy_JA-2-3B'a _miaA | Q2JK09 | WP_011433631.1 | *Synechococcus sp. (strain JA-2-3B'a(2-13))* | 18.5/33.9 |
| Sy_WH7803_miaA | A5GI07 | WP_011932080.1 | *Synechococcus sp. (strain WH7803)* | 19.8/37.4 |
| Te_BP-1_miaA | Q8CWM5 | NP_681437.1 | *Thermosynechococcus elongatus (strain BP-1)* | 19.4/34.9 |
| Te_IMS101_miaA | Q10XN7 | WP_011613317.1 | *Trichodesmium erythraeum (strain IMS101)* | 19.5/37.4 |
| VvIPT1 | GSVIVT01011886001\|Vvinifera | CBI27009.3 | *Vitis vinifera* | 18.1/36.1 |
| VvIPT2 | GSVIVT01014230001\|Vvinifera | CBI20214.3 | *Vitis vinifera* | 16.2/29.4 |
| VvIPT3 | GSVIVT01017885001\|Vvinifera | CBI26180.3 | *Vitis vinifera* | 18.0/33.9 |
| VvIPT4 | GSVIVT01025775001\|Vvinifera | CBI32805.3 | *Vitis vinifera* | 20.7/39.1 |
| VvIPT5 | GSVIVT01031386001\|Vvinifera | CBI17073.3 | *Vitis vinifera* | 14.9/27.6 |
| VvIPT6 | GSVIVT01034295001\|Vvinifera | CBI38978.3 | *Vitis vinifera* | 18.1/34.0 |
| **CKX Sequences** | | | | |
| **Name in tree** | **Name in FASTA** | **GenBank identifier** | **Organism name** | **Identity/Similarity to NoCKX1 (%)** |
| Am_MBIC11017_CKX1 | B0CFD7 | WP_012161407.1 | *Acaryochloris marina (strain MBIC 11017)* | 39.8/60.0 |
| AtCKX1 | AT2G41510.1\|Athaliana | NP_181682.1 | *Arabidopsis thaliana* | 22.4/39.0 |
| AtCKX2 | AT2G19500.1\|Athaliana | NP_565455.1 | *Arabidopsis thaliana* | 26.2/43.4 |
| AtCKX3 | AT5G56970.1\|Athaliana | NP_200507.1 | *Arabidopsis thaliana* | 23.1/39.7 |
| AtCKX4 | AT4G29740.2\|Athaliana | NP_194703.1 | *Arabidopsis thaliana* | 24.4/40.9 |
| AtCKX5 | AT1G75450.1\|Athaliana | NP_001185402.1 | *Arabidopsis thaliana* | 21.9/40.9 |
| AtCKX6 | AT3G63440.1\|Athaliana | NP_191903.3 | *Arabidopsis thaliana* | 22.9/40.2 |
| AtCKX7 | AT5G21482.1\|Athaliana | NP_850863.1 | *Arabidopsis thaliana* | 23.3/41.6 |
| AtrCKX1 | evm_27.model.AmTr_v1.0_scaffold00002.338\|Atrichopoda | XP_006838693.1 | *Amborella trichopoda* | 24.8/41.8 |
| AtrCKX2 | evm_27.model.AmTr_v1.0_scaffold00022.169\|Atrichopoda | XP_006849989.1 | *Amborella trichopoda* | 21.1/34.8 |
| AtrCKX3 | evm_27.model.AmTr_v1.0_scaffold00024.228\|Atrichopoda | XP_006849648.1 | *Amborella trichopoda* | 23.3/41.4 |
| AtrCKX4 | evm_27.model.AmTr_v1.0_scaffold00029.147\|Atrichopoda | XP_006847935.1 | *Amborella trichopoda* | 24.5/43.1 |
| AtrCKX5 | evm_27.model.AmTr_v1.0_scaffold00053.158\|Atrichopoda | XP_006843486.1 | *Amborella trichopoda* | 22.2/40.0 |
| Av_ATCC9413_CKX1 | Q3M3X2\|Avariab | WP_011321379.1 | *Anabaena variabilis (strain ATCC 29413 / PCC 7937)* | 88.1/94.4 |
| No_PCC7120_CKX1 | Q8YZY0 | WP_010994500.1 | *Nostoc sp. (strain PCC 7120 / UTEX 2576)* | 100/100 |
| OsCKX1 | LOC_Os01g09260.1\|Osativa | Q0JQ12.2 | *Oryza sativa* | 21.7/38.5 |
| OsCKX10 | LOC_Os06g37500.1\|Osativa | NP_001055371.2 | *Oryza sativa* | 24.0/40.6 |
| OsCKX11 | LOC_Os08g35860.1\|Osativa | Q6Z955.1 | *Oryza sativa* | 21.9/37.3 |
| OsCKX2 | LOC_Os01g10110.1\|Osativa | Q4ADV8.1 | *Oryza sativa* | 23.9/39.1 |
| OsCKX3 | LOC_Os10g34230.1\|Osativa | NP_001064886.1 | *Oryza sativa* | 21.8/41.3 |
| OsCKX4 | LOC_Os01g71310.1\|Osativa | NP_001045353.1 | *Oryza sativa* | 22.9/40.3 |
| OsCKX5 | LOC_Os01g56810.1\|Osativa | NP_001044409.1 | *Oryza sativa* | 24.0/40.7 |
| OsCKX7 | LOC_Os02g12780.1\|Osativa | Q6YW50.1 | *Oryza sativa* | 25.3/42.8 |
| OsCKX8 | LOC_Os04g44230.1\|Osativa | A2XVN3.1 | *Oryza sativa* | 25.3/42.2 |
| OsCKX9 | LOC_Os05g31040.1\|Osativa | NP_001055371.2 | *Oryza sativa* | 23.8/41.9 |
| PaCKX1 | MA_10432877g0010\|Pabies |  | *Picea abies* | 17.0/30.5 |
| PaCKX2 | MA_113618g0010\|Pabies |  | *Picea abies* | 24.4/41.8 |
| PaCKX3 | MA_138486g0010\|Pabies |  | *Picea abies* | 23.1/40.6 |
| PaCKX4 | MA_14670g0010\|Pabies |  | *Picea abies* | 24.0/39.7 |
| PaCKX5 | MA_17212g0010\|Pabies |  | *Picea abies* | 23.2/41.0 |
| PaCKX6 | MA_31778g0010\|Pabies |  | *Picea abies* | 22.6/38.9 |
| PaCKX7 | MA_32541g0010\|Pabies |  | *Picea abies* | 23.5/40.1 |
| PaCKX8 | MA_42031g0010\|Pabies |  | *Picea abies* | 23.8/39.9 |
| PpCKX1 | Pp1s152_115V6.1\|Ppatens_v1.6 | XP_001772547.1 | *Physcomitrella patens* | 23.2/40.7 |
| PpCKX2 | Pp1s222_68V6.1\|Ppatens_v1.6 | XP_001777489.1 | *Physcomitrella patens* | 22.8/41.3 |
| PpCKX3 | Pp1s595_6V6.1\|Ppatens_v1.6 | XP_001786014.1 | *Physcomitrella patens* | 22.2/38.2 |
| PpCKX4 | Pp1s222_49V6.1\|Ppatens_v1.6 | XP_001777510.1 | *Physcomitrella patens* | 19.4/34.3 |
| PpCKX5 | Pp1s212_108V6.1\|Ppatens_v1.6 | XP_001776926.1 | *Physcomitrella patens* | 19.0/33.6 |
| PpCKX6 | Pp1s403_31V6.1\|Ppatens_v1.6 | XP_001784400.1 | *Physcomitrella patens* | 19.5/36.7 |
| RfCKX1 | RFD188_04925\|Rfascians | WP_032365005.1 | *Rhodococcus fascians* | 27.4/43.8 |
| SmCKX1 | 98722\|Smoellendorffii | XP_002972678.1 | *Selaginella moellendorffii* | 25.0/41.8 |
| SmCKX1 | 98722\|Smoellendorffii | XP_002972678.1 | *Selaginella moellendorffii* | 25.0/41.8 |
| SmCKX2 | 174721\|Smoellendorffii | XP_002975207.1 | *Selaginella moellendorffii* | 23.6/42.4 |
| SmCKX2 | 174721\|Smoellendorffii | XP_002975207.1 | *Selaginella moellendorffii* | 23.6/42.4 |
| VvCKX1 | GSVIVT01001461001\|Vvinifera | CBI28611.3 | *Vitis vinifera* | 20.1/35.1 |
| VvCKX2 | GSVIVT01005041001\|Vvinifera | CBI33379.3 | *Vitis vinifera* | 24.4/40.0 |
| VvCKX3 | GSVIVT01006081001\|Vvinifera | CBI41113.3 | *Vitis vinifera* | 21.4/36.6 |
| VvCKX4 | GSVIVT01006150001\|Vvinifera | CBI33301.3 | *Vitis vinifera* | 24.3/41.4 |
| VvCKX5 | GSVIVT01009786001\|Vvinifera | CBI19763.3 | *Vitis vinifera* | 22.3/39.0 |
| VvCKX6 | GSVIVT01015194001\|Vvinifera | CBI27904.3 | *Vitis vinifera* | 25.5/45.9 |
| VvCKX7 | GSVIVT01028599001\|Vvinifera | CBI37291.3 | *Vitis vinifera* | 26.4/45.2 |
| VvCKX8 | GSVIVT01028610001\|Vvinifera | CBI37295.3 | *Vitis vinifera* | 21.9/38.6 |
| VvCKX9 | GSVIVT01035468001\|Vvinifera | CBI20721.3 | *Vitis vinifera* | 20.9/37.5 |
